# Pseudoangiomatous stromal hyperplasia presenting as accessory axillary breast tissue

**Published:** 2017-07-14

**Authors:** Helen M. Johnson, Tom Reisler

**Affiliations:** ^a^Department of Surgery, Vidant Medical Center, Greenville, NC; ^b^Division of Plastic and Reconstructive Surgery, Department of Surgery, The Brody School of Medicine, East Carolina University, Greenville, NC

**Keywords:** pseudoangiomatous stromal hyperplasia, PASH, breast, benign, hyperplasia

**Figure:**
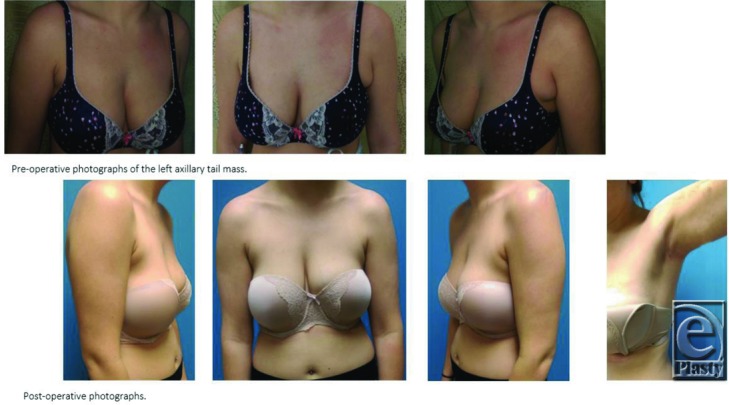


## DESCRIPTION

A 21-year-old woman on oral contraceptives presented with 1-year history of an uncomfortable left axillary mass. The mass had not enlarged and did not vary with menses. No family history of breast cancer was present. Targeted ultrasound scan was read as an 18-mm lymph node. The patient underwent excision, and pathology revealed pseudoangiomatous stromal hyperplasia.

## QUESTIONS

1. **What is pseudoangiomatous stromal hyperplasia (PASH)?**
2. **How does PASH typically present?**
3. **What are the radiographic features of PASH?**
4. **What are the treatment options for PASH?**

## DISCUSSION

PASH is a benign breast lesion first described in 1986 by Vuitch et al.^1^ It is diagnosed histologically and is characterized by dense collagenous stroma interrupted by slit-like spaces. The stromal appearance is due to mesenchymal overgrowth. Most stromal cells express the progesterone receptor. The slit-like spaces resemble blood vessels—hence, the term “angiomatous”—however, they are lined by myofibroblasts rather than endothelial cells, which distinguish PASH from low-grade angiosarcomas.^2^^,^^3^

PASH is rare, with fewer than 200 cases described in the literature.^2^ It can affect both women and men, but the vast majority of cases have been reported in premenopausal women with an average age at diagnosis of 40 years^2^; the male cases have occurred in the context of gynecomastia. PASH is most commonly found incidentally in pathologic specimens, coexisting with either benign or malignant lesions.^3^ PASH can also present as a palpable mass. On examination, tumorous PASH generally presents as a unilateral, firm, well-circumscribed, mobile mass.^2^^,^^3^

PASH does not have any unique radiographic features and often resembles fibroadenoma. On ultrasound scan, PASH generally appears as a well-circumscribed, oval, hypoechoic mass. Mammographically, it is a circumscribed mass without calcifications. On magnetic resonance imaging, it is isointense on T1-weighted images and can appear reticular on T2-weighted images.^2^^,^^3^

There is no well-established treatment algorithm for tumorous PASH. In many cases, the definitive diagnosis is not known until after surgical excision, so it is treated as any other breast mass and worked up with imaging studies and a biopsy. The sensitivity of core-needle biopsy for diagnosing PASH is only 83%,^3^ so biopsies are most useful for determining whether the mass is benign or malignant. Most cases of PASH have been managed with wide local excision, with a recurrence rate of up to 22%.^2^^,^^3^ There have been a few reports of cases refractory to surgical management; in one case, bilateral macromastia recurred 6 months after reduction mammoplasty.^4^ Some argue for nonoperative management with close observation and surveillance imaging, and others advocate for medical management with tamoxifen.^2^^,^^3^

In summary, PASH is an uncommon benign breast lesion that can be found incidentally or present as a mass in premenopausal women or men with gynecomastia. Local excision is the most common treatment, but surveillance and/or hormonal therapy may be appropriate alternatives. Of note, PASH is not associated with an increased risk of malignancy.^5^

## References

[1] Vuitch MF, Rosen PP, Erlandson RA (1986). Pseudoangiomatous hyperplasia of mammary stroma. Hum Pathol.

[2] Jaunoo SS, Thrush S, Dunn P (2011). Pseudoangiomatous stromal hyperplasia (PASH): a brief review. Int J Surg.

[3] Virk RK, Khan A (2010). Pseudoangiomatous stromal hyperplasia: an overview. Arch Pathol Lab Med.

[4] Lee JW, Jung GS, Kim JB (2016). Pseudoangiomatous stromal hyperplasia presenting as rapidly growing bilateral breast enlargement refractory to surgical excision. Arch Plast Surg.

[5] Degnim AC, Frost M, Radisky D (2010). Pseudoangiomatous stromal hyperplasia and breast cancer risk. Ann Surg Oncol.

